# Improving deep learning model performance under parametric constraints for materials informatics applications

**DOI:** 10.1038/s41598-023-36336-5

**Published:** 2023-06-05

**Authors:** Vishu Gupta, Alec Peltekian, Wei-keng Liao, Alok Choudhary, Ankit Agrawal

**Affiliations:** Department of Electrical and Computer Engineering, Northwestern University, Evanston, USA

**Keywords:** Computational methods, Cheminformatics

## Abstract

Modern machine learning (ML) and deep learning (DL) techniques using high-dimensional data representations have helped accelerate the materials discovery process by efficiently detecting hidden patterns in existing datasets and linking input representations to output properties for a better understanding of the scientific phenomenon. While a deep neural network comprised of fully connected layers has been widely used for materials property prediction, simply creating a deeper model with a large number of layers often faces with vanishing gradient problem, causing a degradation in the performance, thereby limiting usage. In this paper, we study and propose architectural principles to address the question of improving the performance of model training and inference under fixed parametric constraints. Here, we present a general deep-learning framework based on branched residual learning (BRNet) with fully connected layers that can work with any numerical vector-based representation as input to build accurate models to predict materials properties. We perform model training for materials properties using numerical vectors representing different composition-based attributes of the respective materials and compare the performance of the proposed models against traditional ML and existing DL architectures. We find that the proposed models are significantly more accurate than the ML/DL models for all data sizes by using different composition-based attributes as input. Further, branched learning requires fewer parameters and results in faster model training due to better convergence during the training phase than existing neural networks, thereby efficiently building accurate models for predicting materials properties.

## Introduction

Modern machine learning (ML) techniques using high-dimensional data representations have seen widespread success in the field of materials science owing to their ability to efficiently detect hidden patterns in existing datasets and link input representations to output properties for a better understanding of the scientific phenomenon and accelerating materials discovery process^1–11^. The process has been catalyzed by the increase in the availability of large-scale datasets through experiments and first-principles calculations such as high throughput density functional theory (DFT) computations^12–17^ and the ease to access and analyze them by using various data mining tools^18,19^. Such application of ML techniques has attracted significant attention throughout the materials science research community and therefore led to the new paradigm of materials informatics^5,20–25^ which has helped materials scientists better understand materials and predict their properties.

Conventionally, traditional ML techniques such as Random Forest, Support Vector Machine, and Decision Tree, have often been applied in materials informatics applications^1–8,26^. Although limited, we have also seen a growing application of more advanced deep learning (DL) techniques in recent years^26–29^. Harvard Energy Clean Project by Pyzer–Knapp et al.^8^ used a three-layer network for predicting the power conversion efficiency of organic photovoltaic materials. Montavon et al.^29^ predicted multiple electronic ground-state and excited-state properties using a model trained on a four-layer network on a database of around 7000 organic compounds. Zhou et al.^27^ used high-dimensional vectors learned using Atom2Vec along with a fully connected network with a single hidden layer to predict formation energy. ElemNet^28^ used a 17-layered architecture to learn formation energy from elemental composition but has shown performance degradation beyond that depth. Some research performed domain knowledge-based model engineering within a deep learning context in materials science for predictive modeling^30–33^. Montavon et al.^26^ trained a four-layer network on a database of around 7000 organic compounds to predict multiple electronic ground-state and excited-state properties. SchNet^30^ incorporated continuous filter convolutional layers to model quantum interactions in molecules for the total energy and inter-atomic forces which follow fundamental quantum chemical principles. CheMixNet^31^ has tried to learn molecular properties from the molecular structures of organic materials by applying deep learning methods. Boomsma and Frellsen introduced the idea of spherical convolution in molecular modeling by making use of the structural environments within proteins. Jha et al.^32^ developed a deep learning framework to predict the crystal orientations of polycrystalline materials from their electron back-scatter diffraction patterns. Work in^33^ performs deep learning by making deeper layered architecture ranging from 10-layer to 48-layer composed of skip connections after every layer using composition and structure based representations to predict materials properties across different datasets. There also have been several efforts to learn either the atomic interaction or the material embeddings using graph-based networks from the crystal structure and composition^34–38^. SchNet is extended in^34^ where the authors used an edge update network to allow for neural message passing between atoms for better property prediction for molecules and materials. Crystal graph convolution neural networks (CGCNN)^35^ directly learn material properties via the connection of atoms in the crystal structure of the crystalline materials, providing an interpretable representation. MatErials Graph Network (MEGNet)^36^ was developed as a universal model for the property prediction of molecules and crystals. Goodall and Lee^37^ developed an architecture that takes stoichiometric attributes instead of crystal attributes as inputs along with matscholar embedding obtained from material science literature using advanced natural language processing algorithms to learn appropriate materials descriptors from data using a graph-based neural network composed of message-passing layer and fully-connected layers. Atomistic Line Graph Neural Network (ALIGNN)^38^ combines atom, bond, and angle-based information obtained from the structure of the materials to obtain high-accuracy models for improved materials property prediction.

In general, introducing complex input attributes, network components, and architecture design has been shown to produce more accurate predictive models for materials properties prediction tasks. However, these improvements require higher computational resources and training time which is undesirable, making it hard to leverage such complex components to build predictive models. Hence, rather than focusing on introducing complex input attributes, network components, and architectural designs in a bid to boost model performance as done in recent works^34–39^, here, we focus on addressing the general issue of how to efficiently build deep neural network architectures for more robust and accurate predictive performance by imposing a parametric constraint (17-layers in our case) and utilizing the available limited computational resources effectively and efficiently. For that, we analyze and propose design principles for a time and parameter-efficient deep learning framework composed of deep neural networks that can predict materials properties using numerical vector-based representations. Since the model architectures for the regression problem are composed of fully connected layers, it is highly non-linear and learning the mapping from input to output is comparatively more challenging than the classification problem. To maximize accuracy and minimize training time under parametric constraints using a neural network composed of fully connected layers, we present a novel approach based on a combination of residual learning with skip connections around a stack of multiple layers ^40–42^ and branched architecture^43–45^, which were originally proposed for classification problems for text or image classification.

We introduce a novel approach to leverage branching in neural networks with and without residual connections for each individual layer (BRNet and BNet). BNet comprises of a series of stacks, each composed of a fully connected layer and LeakyReLU^46^ with a branched structure in the initial layers. BRNet uses BNet as the base network and adds residual connections after each stack for better convergence during the training. BNet and BRNet architectures are designed for the prediction task of learning the formation energy from a vector-based material representation composed of 86 features representing a composition-based elemental fraction as the model input. When trained using $\sim 345$ K samples from the Open Quantum Materials Database (OQMD)^15^, BNet and BRNet achieved a mean absolute error (MAE) of 0.042 eV/atom and 0.041 eV/atom respectively compared to an MAE of 0.149 eV/atom using AutoML^47^. A conference version of this work appeared in Gupta et al.^48^; the current article significantly expands on the conference paper with additional modeling experiments on more datasets, and subsequent analysis of results and insights. We compare our proposed architectures against traditional ML models, and multiple baselines using deep neural network architectures for regression (made using 17 fully connected layers): ElemNet^28^ with dropout at variable intervals of fully connected layers, and individual residual network (IRNet)^33^ with residual connections, batch normalization, and ReLU activation function after each layer. We provide a detailed evaluation and analysis of BNet/BRNet on various publicly available DFT-computed and experimental materials datasets and show that branched networks consistently outperform other ML models and DL networks on the materials property prediction tasks. We also observe that the use of branching leads to faster convergence than existing approaches, while reducing the number of model parameters significantly. BRNet and BNet leverage a simple and intuitive approach of introducing branching with/without residual connections after each layer without using any domain-dependent model engineering, which makes it appealing to researchers working not only on materials but other scientific domains to leverage it for their predictive modeling tasks.

## Results

### Datasets

We use six datasets of DFT-computed and experimental properties in this work: Open Quantum Materials Database (OQMD)^15,49^ with four properties, Automatic Flow of Materials Discovery Library (AFLOWLIB)^50^ with four properties, Materials Project (MP)^14^ with four properties, Joint Automated Repository for Various Integrated Simulations (JARVIS) with five properties^17^, Kingsbury Experimental Formation Enthalpy (KEFE)^51^ with 1 property, and Kingsbury Experimental Band Gap (KEBG)^51^ with 1 property. DFT-computed datasets (OQMD, AFLOWLIB, MP, and JARVIS) were downloaded from the website of the database, and experimental datasets (KEFE and KEBG) were obtained using Matminer^18^. The relevant information about the datasets used to evaluate our methods are shown in Table 1. Please refer to the corresponding publications for further details about each of the listed properties.

**Table 1:** Datasets used in this work.

**Dataset**	**Data size**	**List of properties**
OQMD^15^	345,134	Formation energy (eV/atom), band gap (eV), stability (eV/atom), volume (A$_3$/atom)
AFLOWLIB^50^	234,299	Formation energy (eV/atom), density (grams/cm$_3$), volume (A$_3$/atom), band gap (eV)
MP^14^	89,181	Formation energy (eV/atom), band gap (eV), density (grams/cm$_3$), volume (A$_3$/lattice)
JARVIS^17^	19,994	Formation energy (eV/atom), gap OPT (eV), bulk modulus (GPa), shear modulus (GPa), gap TBMBJ (eV)
Kingsbury experimental formation enthalpy (KEFE)^51^	2135	Formation energy (eV/atom)
Kingsbury experimental band gap (KEBG)^51^	4604	Band gap (eV)

In each of the datasets, materials property values correspond to the lowest formation energy among all compounds with the same composition, representing its most stable crystal structure. The datasets are randomly split with a fixed random seed into training, validation, and test sets in the ratio of 81:9:10.

### Model architecture design

Many of the existing DL works in materials science focus on introducing complex input attributes, network components, and architectural designs to boost model performance^34–39^. Given that computational resources are usually limited, and oftentimes we only see marginal improvements in the accuracy of the model as compared to the exponential increase in the number of parameters added to the architecture of the deep neural network^33^, analyzing design principle to improve the accuracy of the model under parametric constraints might be a more practical and useful goal to work towards. We thus explore a novel approach of using branching at the early stage of the deep neural network architecture composed of fully connected layers to maximize the performance of the model under a parametric constraint. Note that parametric constraint in this work refers to using a fixed number of layers for constructing the architecture of the deep neural network, i.e., 17 layers in our case. We design two deep neural networks (BRNet and BNet) which contain branching with/without residual connections where both the proposed networks take a numerical vector-based representation as model input to predict the materials property of interest.

BRNet and BNet architectures are designed for the prediction task of learning the formation energy from a numerical vector-based representation composed of 86 features representing a composition-based elemental fraction as the model input. The deep neural network architectures are composed of fully connected layers, where each fully connected layer is followed by LeakyReLU^46^ as the activation function with (BRNet) and without (BNet) residual connections. To demonstrate the impact of our approach, we compare our proposed architectures against traditional ML models and multiple existing architectures (ElemNet and IRNet) comprised of the same number of layers (17 fully connected layers in our case) for fair comparison in terms of parametric constraint. In this study, we give ElemNet and IRNet architecture different sets of inputs for model training than what was previously used in their respective works to test the generalized performance of the different architectures. For a detailed description of the existing architectures (ElemNet and IRNet), the reader is referred to their respective publications^28,33^. We show the performance comparison of the proposed architectures with other existing deep neural networks for formation energy as the materials property and composition-based elemental fraction as the model input using various datasets in Table 2.

**Table 2:** Test MAE of different models for the prediction task of “Model Architecture Design”.

**Dataset**	**Size**	**AutoML**	**ElemNet**	**IRNet**	**BNet**	**BRNet**
OQMD	345,134	0.149	0.049	0.042	0.042	**0.041**
AFLOWLIB	234,299	0.115	0.058	0.051	0.048	**0.047**
MP	89,181	0.167	0.121	0.117	0.112	**0.106**
JARVIS	19,994	0.129	0.083	0.094	0.071	**0.070**

Here, we use formation energy (eV/atom) as the materials property and composition-based elemental fraction as the model input.

The lowest MAE values in each row are highlighted in bold.

Table 2 shows that the proposed architectures significantly outperform the traditional ML models for all the datasets. We also trained existing deep neural network architectures on this prediction task and observed that branching significantly reduces the prediction error, which illustrates its benefit over traditional ML models, ElemNet, and IRNet for the design task. We observe a relatively small difference between the accuracy of BNet and BRNet models, which is due to the presence of residual connections that help prevent vanishing and/or exploding gradient issues for deep neural network architectures.

### Other materials properties

Next, we demonstrate the significance of branching on the prediction modeling tasks of “Other materials properties”. We train BRNet and BNet for predicting materials properties from numerical vector-based representation composed of 86 features representing composition-based elemental fractions as the model input. To illustrate the impact of branching, we also compare the performance of our proposed networks against traditional ML algorithms, ElemNet, and IRNet.

**Table 3:** Test MAE of different models for each of the materials properties for the prediction task of “Other materials properties”.

**Dataset**	**Property**	**Size**	**AutoML**	**ElemNet**	**IRNet**	**BNet**	**BRNet**
OQMD	Band gap (eV)	345,134	0.075	0.052	0.054	0.050	**0.048**
	Stability (eV/atom)	345,134	0.113	0.051	0.047	0.045	**0.043**
	Volume (A$_3$/atom)	345,134	21.02	19.56	20.09	17.92	**16.91**
AFLOWLIB	Density (grams/cm$_3$)	234,299	0.556	0.227	0.186	0.184	**0.176**
	Volume (A$_3$/atom)	234,299	1.001	0.690	0.611	0.603	**0.588**
	Band gap (eV)	14,751	0.134	0.145	0.140	0.116	**0.108**
MP	Band gap (eV)	89,181	0.435	0.342	0.316	0.317	**0.315**
	Density (grams/cm$_3$)	89,181	0.446	0.373	0.373	0.349	**0.344**
	Volume (A$_3$/lattice)	89,181	**205.9**	248.8	238.6	233.8	227.4
JARVIS	Gap OPT (eV)	17,924	0.345	0.294	0.300	0.265	**0.260**
	Bulk modulus (GPa)	8199	13.46	11.56	11.71	11.79	**10.63**
	Shear modulus (GPa)	8199	10.75	10.64	10.75	11.10	**9.94**
	Gap TBMBJ (eV)	5287	0.630	0.544	0.526	**0.483**	0.497

The lowest MAE values in each row are highlighted in bold.

**Figure 1:**
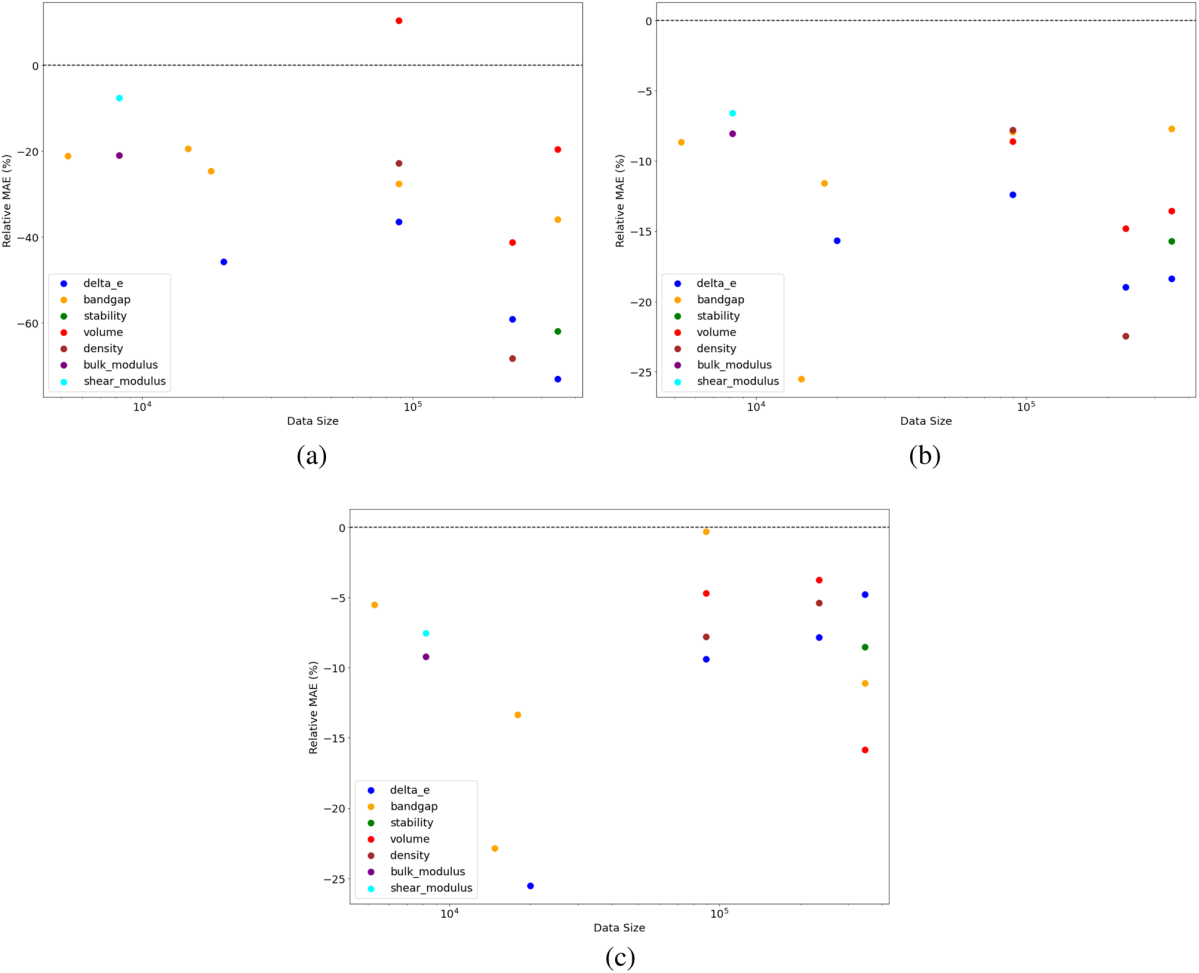
The figure indicates the percentage change in test MAE of the proposed BRNet w.r.t (**a**) AutoML, (**b**) ElemNet, and (**c**) IRNet. The x-axis shows the dataset size on a log scale, and the y-axis shows the percentage change in test MAE from all the model training performed in Tables 2 and 3 calculated as ((MAE$$_{BRNet}$$/MAE$$_{Other})-1$$) $$\times$$ 100$$\%$$.

We observe in Table 3 that the branched architectures BRNet and BNet almost always outperform other DL models and traditional ML algorithms. The performance of traditional ML algorithms is always the worst, except for one case where it outperformed other models. Among the branching architectures, BRNet outperforms BNet for model training using composition-based elemental fraction as the model input in almost all cases since the BNet does not have any residual connections, which makes it susceptible to performance degradation issues due to vanishing and/or exploding gradients. BRNet significantly benefits from the use of residual connections, which helps with smooth gradient flow during backpropagation. For JARVIS with Gap TBMBJ as materials property and data size < 5300, we find that BNet performs better than BRNet. We also plot the percentage change in test MAE of the proposed BRNet w.r.t other pre-existing models i.e. AutoML, ElemNet, and IRNet in Fig. 1.

Figure 1 shows that BRNet outperforms the traditional ML algorithms (with up to 70% reduction in MAE) and existing DL models (with up to 25% reduction in MAE) with the same number of layers in the architecture for almost all materials properties in the four datasets used in this performance evaluation analysis. This clearly illustrates the benefit of leveraging the concept of branching for the given prediction task of “Other materials properties”. After this exploration, we exclude AutoML from further analysis as it is found to not benefit much for this problem.

### Other materials representation

Next, we illustrate the versatility of leveraging branching in the deep neural network architecture by building models with different composition-based attributes as model input. We train BRNet, BNet, ElemNet, and IRNet similar to the previous analysis, but use 145 composition-based physical attributes^3^ for model input instead of 86 elemental fractions (EF)^28^. Table 4 demonstrates the performance of proposed models using different types of materials representation in the model input for datasets with various sizes.

**Table 4:** Test MAE of different models for each of the materials properties for the prediction task of “Other materials representation”.

**Dataset**	**Property**	**Size**	**ElemNet**	**IRNet**	**BNet**	**BRNet**
OQMD	Formation energy (eV/atom)	345,134	0.084	0.062	0.053	**0.051**
	Band gap (eV)	345,134	0.065	0.050	**0.049**	0.051
	Stability (eV/atom)	345,134	0.073	0.064	**0.053**	**0.053**
	Volume (A$_3$/atom)	345,134	18.4388	17.02	**16.00**	16.52
AFLOWLIB	Formation energy (eV/atom)	234,299	0.069	0.061	**0.050**	0.051
	Density (grams/cm$_3$)	234,299	0.226	0.187	0.188	**0.186**
	Volume (A$_3$/atom)	234,299	0.723	0.629	0.628	**0.612**
	Band gap (eV)	14,751	0.155	0.114	**0.109**	0.114
MP	Formation energy (eV/atom)	89,181	0.158	0.143	0.141	**0.133**
	Band gap (eV)	89,181	0.358	0.335	**0.334**	0.342
	Density (grams/cm$_3$)	89,181	0.410	0.363	0.362	**0.361**
	Volume (A$_3$/lattice)	89,181	234.1	227.2	**226.4**	231.4
JARVIS	Formation energy (eV/atom)	19,994	0.126	0.140	**0.104**	**0.104**
	Gap OPT (eV)	17,924	0.296	0.309	**0.284**	0.294
	Bulk modulus (GPa)	8199	12.33	12.19	11.97	**11.79**
	Shear modulus (GPa)	8199	11.01	10.58	10.54	**10.39**
	Gap TBMBJ (eV)	5287	0.619	0.537	0.566	**0.531**

The lowest MAE values in each row are highlighted in bold.

**Figure 2:**
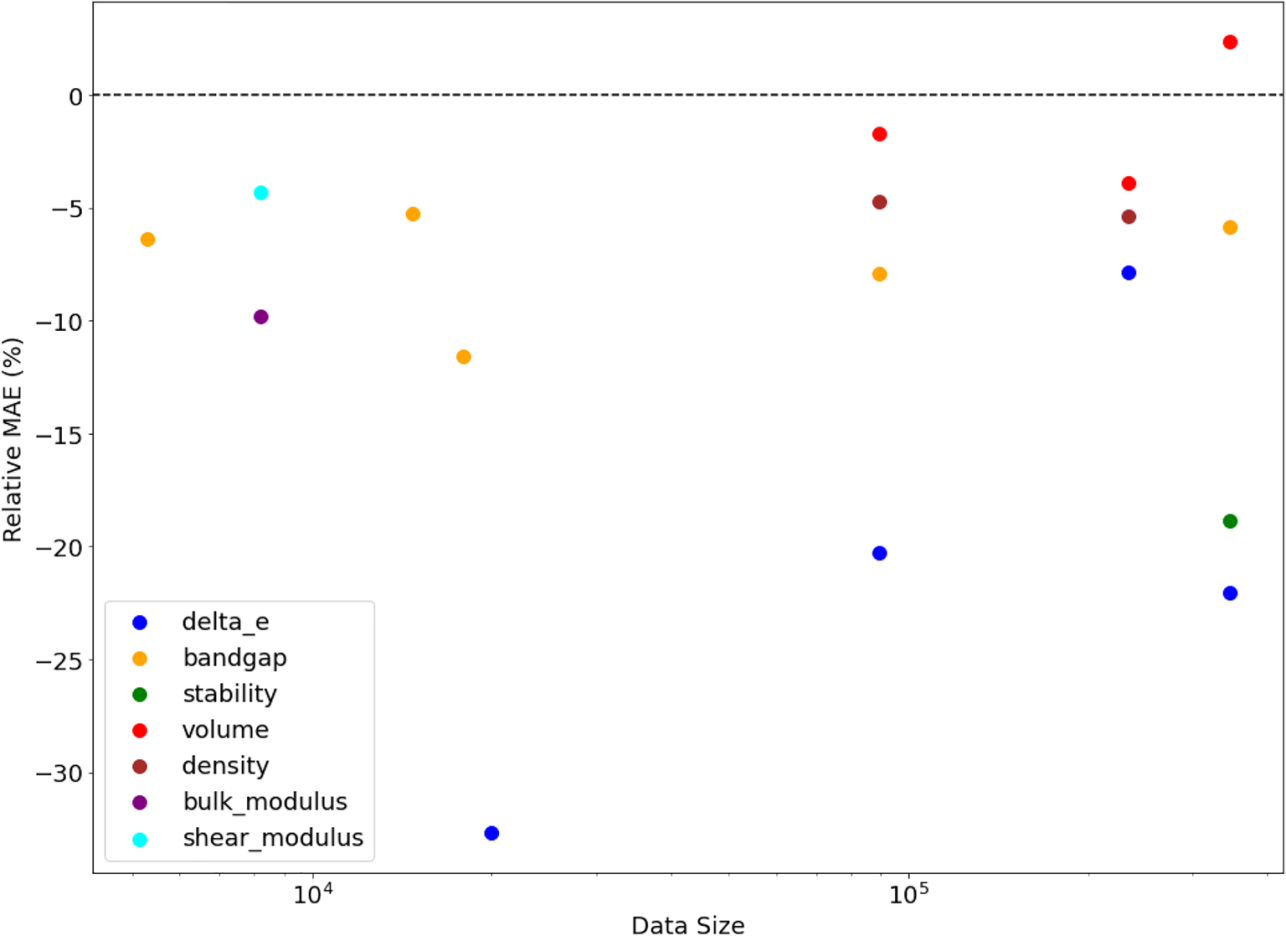
Impact of input representation on the accuracy of BRNet. The x-axis shows the dataset size on a log scale, and the y-axis shows the percentage change in MAE of the model trained using composition-based elemental fraction as input w.r.t. the model trained using composition-based physical attributes as input (calculated as ((MAE$$_{EF}$$/MAE$$_{PA})-1$$) $$\times$$ 100$$\%$$).

From Table 4, we find that our proposed networks perform better as compared to other DL models in all the datasets which shows that the approach involving branching of the deep neural network architecture significantly helps in accurately learning the materials properties from the given materials representations as compared to other DL networks. An interesting observation from Table 4 is that the number of cases for which BNet performs the best is almost equal to that of BRNet which shows that depending on the type of input provided to the branched architecture, the presence of residual connection may not always contribute towards further enhancing the accuracy of the model. This illustrates the versatility of leveraging branched deep neural network architecture for the general prediction modeling task of materials property given any type of vector-based materials representation. Additionally, we analyze the impact of the input representation used for model training on the accuracy of the model by comparing the composition-based elemental fraction and composition-based physical attributes using BRNet in Fig. 2. Interestingly, we observe that numerical vector-based representation composed of composition-based elemental fraction performs better as compared to the composition-based physical attributes. We believe this might be due to the widely recognized ability of deep neural networks to work well on raw inputs without any feature engineering^28,52^. Hence, we will only use the numerical vector-based representation composed of composition-based elemental fractions for further analysis.

### Performance on experimental datasets

In our analysis, we generally observe the benefit of leveraging branched deep neural network architecture which tends to perform better than other DL networks and traditional ML models. Here, we investigate the performance of the proposed networks against the experimental datasets which are usually small in size as compared to the DFT-computed datasets. We train traditional ML models and DL models using numerical vector-based representation composed of 86 features representing composition-based elemental fractions as the model input.

**Table 5:** Test MAE of different models for each of the materials properties for the prediction task of “Performance on Experimental Datasets”.

**Dataset**	**Property**	**Size**	**AutoML**	**ElemNet**	**IRNet**	**BNet**	**BRNet**
KEFE	Formation enthalpy (eV/atom)	2135	0.189	0.129	0.123	0.103	**0.096**
KEBG	Band gap (eV)	4604	0.464	0.468	0.493	0.483	**0.460**

The lowest MAE values in each row are highlighted in bold.

From Table 5, we observe similar trends as our previous analysis where the proposed architectures outperform the traditional ML models and existing DL models with the same number of layers in the architecture for both the experimental datasets in this analysis. We believe this will motivate materials scientists to leverage branched architecture to build their deep neural network architectures for materials property prediction tasks.

### Performance analysis

Next, we perform performance analysis using a bubble chart, prediction error chart, and cumulative distribution function (CDF) of the prediction errors. We mainly compare the accuracy and training time of different deep neural networks comprised of the same number of layers when trained using numerical vector-based representation composed of composition-based elemental fractions on formation energy from four different DFT-computed datasets (OQMD, AFLOWLIB, MP, and JARVIS).

**Figure 3:**
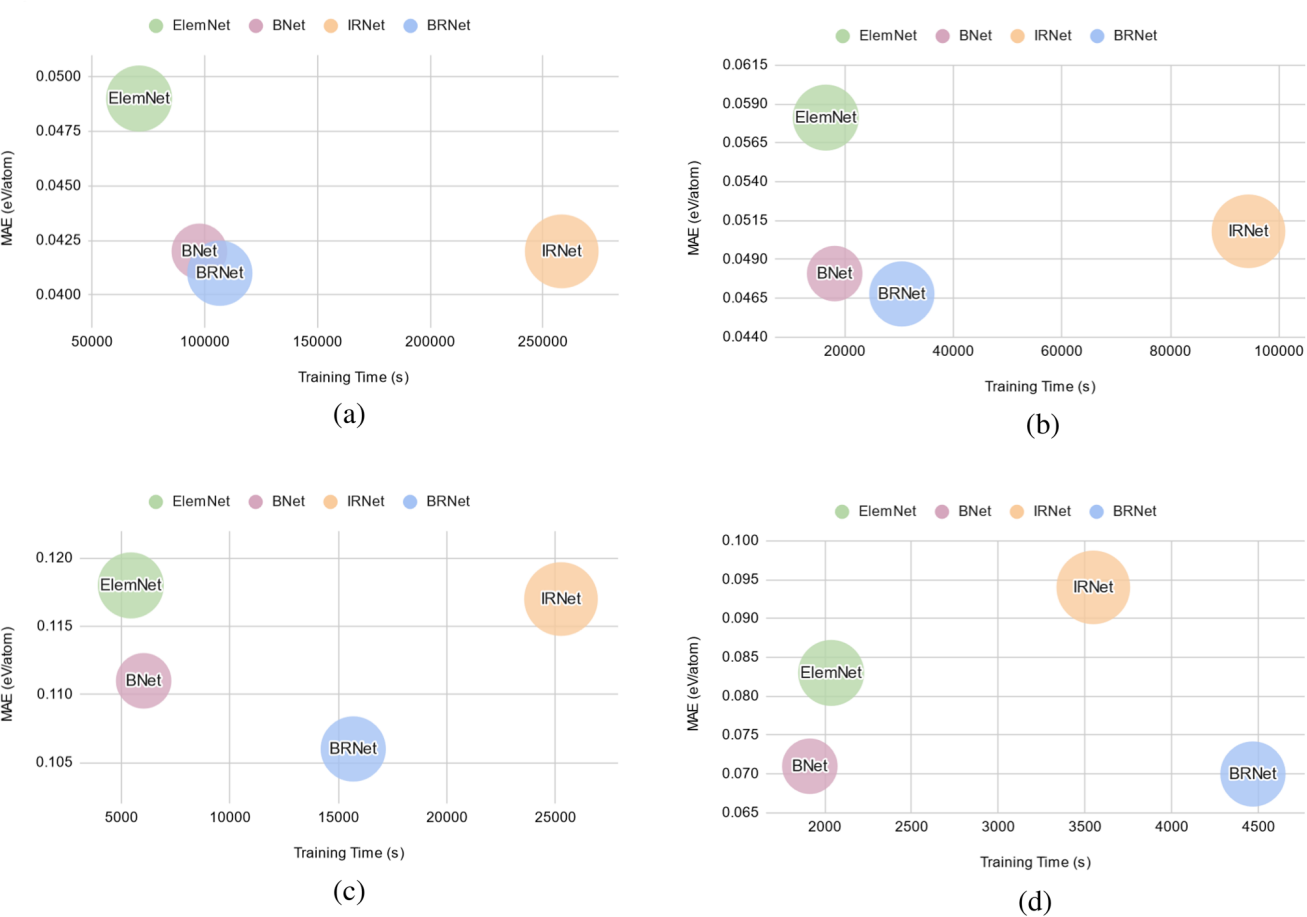
The bubble charts indicate the performance of the DL models based on the training time on the x-axis, MAE (eV/atom) on the y-axis, and the number of model parameters as the bubble size for (**a**) OQMD, (**b**) AFLOWLIB, (**c**) MP, and (**d**) JARVIS. The bubbles closer to the bottom-left corner of the chart correspond to less training time as well as low MAE, and thus are desirable.

Figure 3 shows the bubble charts that indicate the performance in terms of training time on the x-axis, MAE on the y-axis, and bubble size as the model parameters for different DL models using formation energy of the four DFT-computed datasets as the materials property. The closer the DL model is to the bottom-left corner of the bubble chart, the better the overall performance is of that model, as it is able to train faster and produce an accurate model. We observe the following trends from Fig. 3: (1) ElemNet architecture takes less training time but produces a less accurate model for almost all the cases. For the JARVIS dataset (which is comparatively smaller in size) it is able to outperform IRNet in terms of accuracy, but it still is not the fastest or the most accurate model; (2) IRNet architecture, in general, takes more time to train the model, but that training time is not always translated into high accuracy of the model. This also suggests that for the regression-based materials property prediction task, the presence of batch normalization as one of the components of the deep neural network architecture might not be helpful for significantly improving the accuracy of the model, while also keeping the training time reasonably low; (3) The proposed branched deep neural network architectures are almost always closer to the bottom-left corner of the bubble chart, with BNet usually slightly faster in terms of training time and BRNet slightly better in terms of accuracy. For the JARVIS dataset, BRNet took a lot of time to train the model, but it produced the most accurate model, which can be beneficial if the main objective is to build the most accurate model under a fixed parametric constraint.

**Figure 4:**
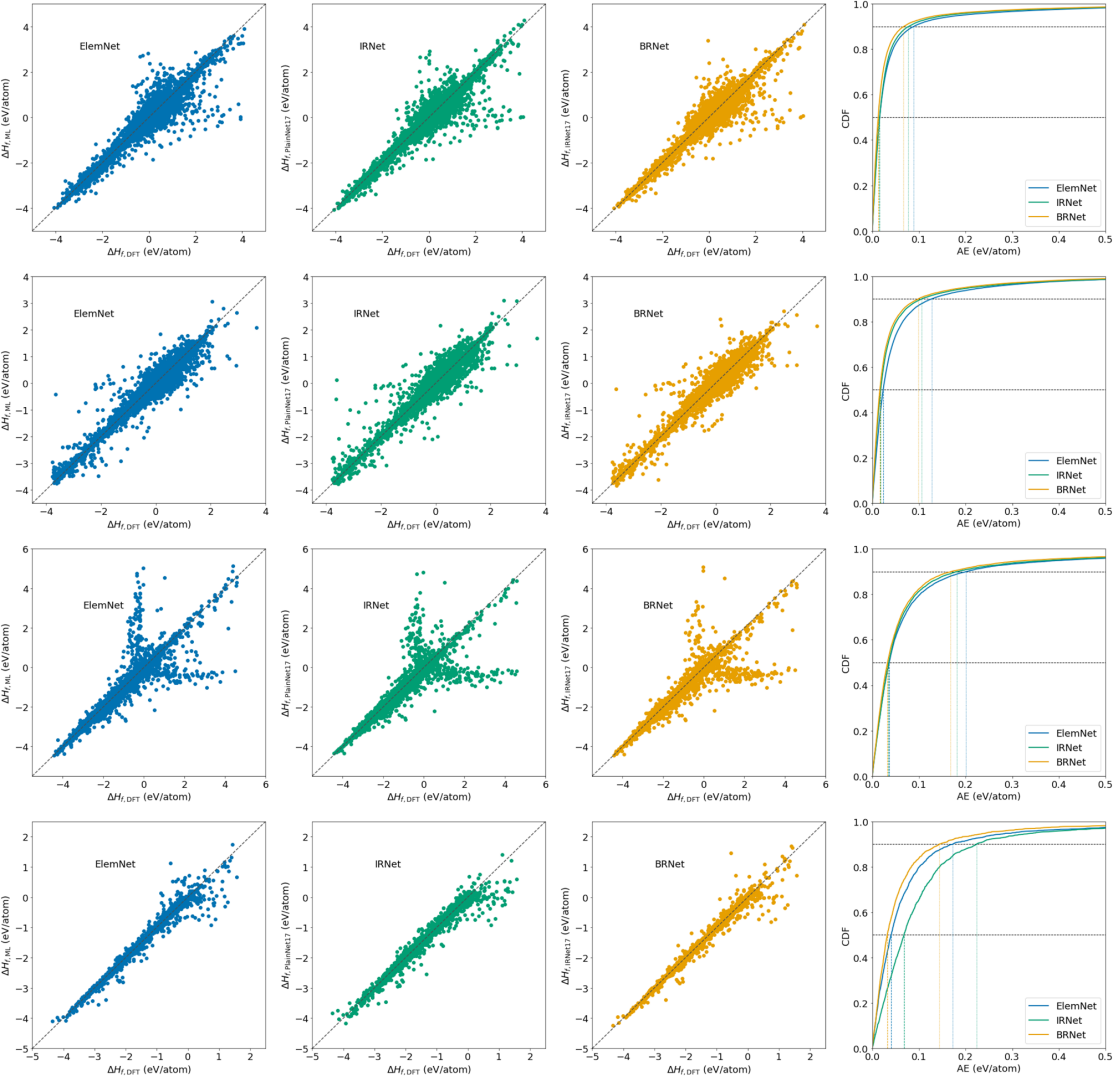
Comparison of ElemNet, IRNet against proposed BRNet on formation energy of four different DFT-computed datasets using composition-based elemental fractions as model inputs. The rows represent different DFT-computed datasets in the order of OQMD, AFLOWLIB, MP, and JARVIS from top to bottom. Within each row, the first three subplots represent the prediction errors using three models: ElemNet, IRNet, and BRNet; the last subplot contains the cumulative distribution function (CDF) of the prediction errors using the three models, with 50th and 90th percentiles marked.

Figure 4 illustrates the prediction error distribution for formation enthalpy in four DFT-computed datasets using composition-based elemental fraction as model input. Although the scatter plot of ElemNet, IRNet and BRNet look similar, we can observe that the prediction moves closer to the diagonal for BRNet. Scatter plots also illustrate that all three models have outliers, with outliers in the case of BRNet being relatively closer to the diagonal. The difference in prediction error distributions becomes more evident from the CDF (cumulative distributive function) curves for the three models, where we observe that the 90th percentile absolute prediction error for BRNet is lower than ElemNet and IRNet for all four DFT-computed datasets. The performance analysis demonstrates the advantage of leveraging branched deep neural network architecture for given materials representations as the model input for better prediction performance under a fixed parametric constraint.

## Discussion

We presented a novel approach to leverage the concept of branching in deep neural network architecture to enable better performance for materials property prediction under parametric constraints. To illustrate the benefit of leveraging the proposed approach, we built a general deep learning framework composed of branched deep neural network architectures BRNet and BNet. To compare the performance of the proposed models, we use traditional ML algorithms and existing deep neural networks ElemNet and IRNet, which consist of the same number of layers in their architecture to ensure a fair comparison of parametric constraints. The proposed BRNet and BNet architectures were designed (optimized) for the task of predicting formation energy using a numerical vector-based representation composed of 86 composition-derived elemental fractions as the model input. On the design problem, the proposed models leveraging the proposed design approach significantly outperformed the traditional ML algorithms, ElemNet and IRNet. We demonstrated the efficacy of the proposed approach by evaluating and comparing these DL model architectures against ElemNet, IRNet, and traditional ML algorithms on a variety of materials properties available across multiple materials datasets. Furthermore, we demonstrated that the presented DL model architectures leveraging the proposed approach are versatile in their vector-based model input by evaluating prediction models for different materials properties using different numerical vector-based representations, i.e., composition-derived 145 physical attributes and composition-derived 86 elemental fractions. The proposed approach outperforms other ML/DL models in terms of model accuracy irrespective of the size of the materials property being analyzed, where branching provides a better capability to capture the mapping between the given input material representation and the output property. In general, the training time of a deep neural network model depends on the given prediction task (model inputs and model output), the size of the training dataset, and the architecture and depth of the neural networks (number of model parameters). In our case, as the depth of the neural networks (the number of layers used to construct the architecture) is fixed, the complexity and components used to construct the architecture is the only factor that can affect the training time. We see that the use of branched architecture helps in a significant reduction of training time as compared to other baseline architectures used for comparison. Additionally, to check the robustness of the proposed branched architectures even further, we perform empirical and statistical analysis to explore the benefits of branching in deep neural networks. In the empirical analysis, we perform predictive analysis by changing the location of the branch and the number of branches for a given location under fixed parametric constraints (i.e., 17 layers in our case as depicted in Supplementary Fig. 1) using the formation energy of various datasets as the materials property which is shown in Table 6. We limit our analysis to a single occurrence of branching, which can be configured at multiple locations in various distributions.

**Table 6:** Effect of branching on model accuracy and parameters by changing the location and distribution of branching for a single occurrence of branching.

Branching	Branching	$\#$Model	$\#$Model	Dataset			
Location	Distribution	Size (MiB)	Parameters	OQMD	AFLOWLIB	MP	JARVIS
Top	[2, 2]	18.3	4,548,385	0.041	0.048	0.106	0.070
	[3, 1]	18.3	4,548,385	0.043	0.049	0.113	0.069
	[2, 1, 1]	14.8	3,676,961	0.041	0.048	0.107	0.068
	[1, 1, 1, 1]	11.3	2,805,537	0.041	0.047	0.108	0.069
Middle 1	[2, 1]	24.9	6,206,753	0.041	0.048	0.113	0.068
	[1, 1, 1]	28.1	6,993,697	0.041	0.048	0.111	0.070
Middle 2	[2, 1]	22.6	5,616,673	0.040	0.048	0.111	0.069
	[1, 1, 1]	23.4	5,813,537	0.042	0.048	0.113	0.071
Middle 3	[2, 1]	22.0	5,469,089	0.041	0.049	0.108	0.069
	[1, 1, 1]	22.2	5,518,369	0.041	0.049	0.114	0.070
Bottom	[1, 1]	21.8	5,432,161	0.041	0.047	0.112	0.067

Table 6 shows the effect of branching on model accuracy and parameters by changing the location and distribution of branching for a single occurrence of branching. We observe that changing the configuration of the branching does not significantly vary the performance of the model for large datasets. For small datasets, the variation in model accuracy is slightly higher, which is not surprising. We also observe that branching at the initial layers of the neural network or for the layers with a large number of neurons and increasing the number of branches under parametric constraint decreases the model size and number of model parameters without significant change in the accuracy of the model. More sophisticated branching with simultaneous multiple branching at different locations with/without increasing the number of layers would be an interesting future study.

Next, we perform statistical analysis where we estimate a one-tailed *p*-value to compare the test MAEs obtained using the 5 $\times$ 2-fold cross-validation (5 $\times$ 2 CV) of formation energy of four datasets (OQMD, AFLOWLIB, MP, and JARVIS) as the materials property in order to see if the observed improvement in accuracy of the proposed BNet/BRNet over existing models is significant or not. The mean ± standard deviation of the test MAE for the 5 $\times$ 2 CV is shown in Supplementary Table 2. We use the corrected paired t-test proposed by Nadeau and Bengio^53^ to estimate the one-tailed *p*-value. Here, the null hypothesis is “BNet/BRNet models are worse than the existing models” and the alternate hypothesis is “BNet/BRNet models are better than the existing models”. After performing the statistical testing using the corrected paired t-test, we get the *p*-value < 0.05 (for both the comparisons, i.e., BNet or BRNet against existing models), thus rejecting the null hypothesis at $\alpha$ = 0.05. This suggests that the difference in test MAE between BNet/BRNet and existing models is unlikely to have arisen by chance, and thus we can infer that in general, the proposed BNet/BRNet models perform significantly better than existing models. We also calculate the one-tailed *p*-value to compare the test MAEs of BNet and BRNet and obtain the *p*-value < 0.05 for 3 out of 4 cases. For the MP dataset, although BNet performed better as compared to BRNet in terms of mean ± standard deviation of the test MAE, we obtained the *p*-value > 0.05, which shows that the results are not significantly better statistically. This shows that, in general, BRNet tends to perform at least comparable or better than BNet. Since the proposed approach of leveraging branching the deep neural network architecture in BRNet and BNet does not depend on any particular material representation/embedding as model input, we expect that it can also be used to improve the performance of other DL works leveraging other types of materials representations in materials science and other scientific domains. The proposed approach of branched deep neural network architecture is conceptually simple to implement and build upon. The BRNet framework code is publicly available at https://github.com/GuptaVishu2002/BRNet.

## Methods

### Model architectures

**Figure 5:**
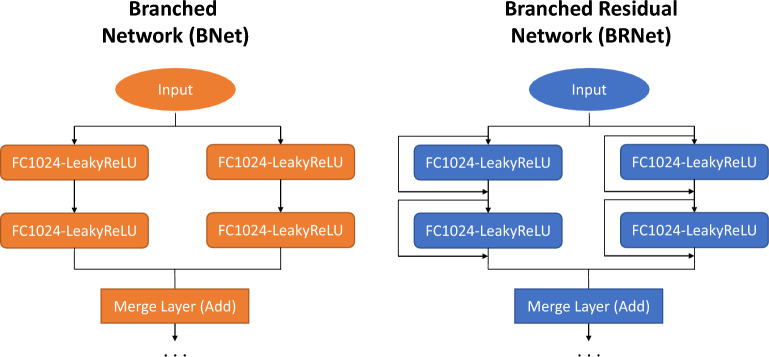
Design approach for BNet (left) and BRNet (right). The proposed approach of branched deep neural network architecture has branching of the layers at the initial layers with and without residual connection around each layer to make it easy for the model to learn the mapping of output materials property from a given materials representation as the model input.

The design approach and mathematical formulation for branched deep neural network architecture is illustrated in Fig. 5 and supplementary information. The model architecture is formed by putting together a series of stacks, each composed of one or more sequences of two basic components with the same configuration. Since we use numerical vector-based representation as model input (refer to supplementary information for a detailed description of the model inputs), the model uses a fully connected layer as the initial layer in each sequence which is followed by LeakyReLU^46^ as the activation function. The simplest instantiation of this architecture adds no residual connections and thus learns simply the approximate mapping from input to output which we refer to as Branched Network (BNet). We also create a deep neural network architecture with residual connection after every sequence, so that each sequence needs only to learn the residual mapping between its input and output. The residual connection has the effect of making the regression learning task easier and providing a smooth flow of gradients between layers. We refer to this deep neural network as a branched residual network (BRNet). The implementation of all the models used in this work is publicly available at https://github.com/GuptaVishu2002/BRNet.

### Network and ML settings

We implement the deep learning models with Python and TensorFlow 2^54^ and Keras^55^. We found the best hyperparameters to be Adam^56^ as the optimizer with a mini-batch size of 32, alearning rate of 0.0001, mean absolute error as loss function, and LeakyReLU^46^ as activation function after every fully connected layer (except for the final layer which has no activation function). Rather than training the model for a specific number of epochs, we used early stopping with patience of 100 epochs, meaning that we stopped training when the performance did not improve in 100 epochs. For traditional ML models, we used an AutoML library called hyperopt sklearn^47^ to find the best-performing ML model implementations and employed mean absolute error (MAE) as loss function and error metric. For the number of model parameters and model size used by each of the deep learning models, please refer to Supplementary Table 1.

## Supplementary Information

Supplementary Information.

## Data Availability

All the datasets used in this paper are publicly available from their corresponding websites- OQMD (http://oqmd.org), AFLOWLIB (http://aflowlib.org), Materials Project (https://materialsproject.org), JARVIS (https://jarvis.nist.gov), and using Matminer (https://hackingmaterials.lbl.gov/matminer/).
