# Supplementary material for: Improving deep learning model performance under parametric constraints for materials informatics applications
Source: Sci Rep. 2023 Jun 5;13:9128. doi: 10.1038/s41598-023-36336-5 (PMC10241826; doi:10.1038/s41598-023-36336-5)
Supplement: Supplementary file 1 — Supplementary Information. [file 41598_2023_36336_MOESM1_ESM.pdf]

Supplementary Information: Improving Deep Learning Model Performance under Parametric Constraints for Materials Informatics Applications

Vishu Gupta[1], Alec Peltekian[1], Wei-keng Liao[1], Alok Choudhary[1], Ankit Agrawal[1]

[1]*Department of Electrical and Computer Engineering, Northwestern University*

Model Inputs

Elemental Fractions Elemental fractions [1] (EF) is composed of 86 composition-based numerical vectors where each attribute represents an element in the periodic table. For example, an arbitrary compound $X_aY_bZ_c$ (where X, Y, and Z are elements of the periodic table) is represented as a 1D vector of 86 numbers, with the X, Y, and Z element columns containing $a/(a+b+c)$, $b/(a+b+c)$, and $c/(a+b+c)$ respectively and everything else as 0. The list of all the elements used in EF is as follows:

H, Li, Be, B, C, N, O, F, Na, Mg, Al, Si, P, S, Cl, K, Ca, Sc, Ti, V, Cr, Mn, Fe, Co, Ni, Cu, Zn, Ga, Ge, As, Se, Br, Kr, Rb, Sr, Y, Zr, Nb, Mo, Tc, Ru, Rh, Pd, Ag, Cd, In, Sn, Sb, Te, I, Xe, Cs, Ba, La, Ce, Pr, Nd, Pm, Sm, Eu, Gd, Tb, Dy, Ho, Er, Tm, Yb, Lu, Hf,

Ta, W, Re, Os, Ir, Pt, Au, Hg, Tl, Pb, Bi, Ac, Th, Pa, U, Np, Pu

Physical Attributes Physical attributes [2] (PA) are composed of 145 compositions (MAGPIE) based numerical vectors where the attributes represent stoichiometric attributes, elemental property statistics, electronic structure attributes, and ionic compound attributes. All the attributes are obtained using the featurization functionality available in Matminer [3]. The list of all the attributes used in PA is as follows:

0-norm, 2-norm, 3-norm, 5-norm, 7-norm, 10-norm, MagpieData minimum Number, MagpieData maximum Number, MagpieData range Number, MagpieData mean Number, MagpieData avg-dev Number, MagpieData mode Number, MagpieData minimum MendeleevNumber, MagpieData maximum MendeleevNumber, MagpieData range Mendeleev Number, MagpieData mean MendeleevNumber, MagpieData avg-dev MendeleevNumber, MagpieData mode MendeleevNumber, MagpieData minimum AtomicWeight, MagpieData maximum AtomicWeight, MagpieData range AtomicWeight, MagpieData mean AtomicWeight, MagpieData avg-dev AtomicWeight, MagpieData mode AtomicWeight, MagpieData minimum MeltingT, MagpieData maximum MeltingT, MagpieData range MeltingT, MagpieData mean MeltingT, MagpieData avg-dev MeltingT, MagpieData mode MeltingT, MagpieData minimum Column, MagpieData maximum Column, MagpieData range Coalumn, MagpieData mean Column, MagpieData avg-dev Column, MagpieData

mode Column, MagpieData minimum Row, MagpieData maximum Row, MagpieData range Row, MagpieData mean Row, MagpieData avg-dev Row, MagpieData mode Row, MagpieData minimum CovalentRadius, MagpieData maximum CovalentRadius, MagpieData range CovalentRadius, MagpieData mean CovalentRadius, MagpieData avg-dev CovalentRadius, MagpieData mode CovalentRadius, MagpieData minimum Electronegativity, MagpieData maximum Electronegativity, MagpieData range Electronegativity, MagpieData mean Electronegativity, MagpieData avg-dev Electronegativity, MagpieData mode Electronegativity, MagpieData minimum NsValence, MagpieData maximum NsValence, MagpieData range NsValence, MagpieData mean NsValence, MagpieData avg-dev NsValence, MagpieData mode NsValence, MagpieData minimum NpValence, MagpieData maximum NpValence, MagpieData range NpValence, MagpieData mean NpValence, MagpieData avg-dev NpValence, MagpieData mode NpValence, MagpieData minimum NdValence, MagpieData maximum NdValence, MagpieData range NdValence, MagpieData mean NdValence, MagpieData avg-dev NdValence, MagpieData mode NdValence, MagpieData minimum NfValence, MagpieData maximum NfValence, MagpieData range NfValence, MagpieData mean NfValence, MagpieData avg-dev NfValence, MagpieData mode NfValence, MagpieData minimum NValence, MagpieData maximum NValence, MagpieData range NValence, MagpieData mean NValence, MagpieData avg-dev NValence, MagpieData mode NValence, MagpieData minimum NsUnfilled, MagpieData maximum

NsUnfilled, MagpieData range NsUnfilled, MagpieData mean NsUnfilled, MagpieData avg-dev NsUnfilled, MagpieData mode NsUnfilled, MagpieData minimum NpUnfilled, MagpieData maximum NpUnfilled, MagpieData range NpUnfilled, MagpieData mean NpUnfilled, MagpieData avg-dev NpUnfilled, MagpieData mode NpUnfilled, MagpieData minimum NdUnfilled, MagpieData maximum NdUnfilled, MagpieData range NdUnfilled, MagpieData mean NdUnfilled, MagpieData avg-dev NdUnfilled, MagpieData mode NdUnfilled, MagpieData minimum NfUnfilled, MagpieData maximum NfUnfilled, MagpieData range NfUnfilled, MagpieData mean NfUnfilled, MagpieData avg-dev NfUnfilled, MagpieData mode NfUnfilled, MagpieData minimum NUnfilled, MagpieData maximum NUnfilled, MagpieData range NUnfilled, MagpieData mean NUnfilled, MagpieData avg-dev NUnfilled, MagpieData mode NUnfilled, MagpieData minimum GSvolume-pa, MagpieData maximum GSvolume-pa, MagpieData range GSvolume-pa, MagpieData mean GSvolume-pa, MagpieData avg-dev GSvolume-pa, MagpieData mode GSvolume-pa, MagpieData minimum GSbandgap, MagpieData maximum GSbandgap, MagpieData range GSbandgap, MagpieData mean GSbandgap, MagpieData avg-dev GSbandgap, MagpieData mode GSbandgap, MagpieData minimum GSmagmom, MagpieData maximum GSmagmom, MagpieData range GSmagmom, MagpieData mean GSmagmom, MagpieData avg-dev GSmagmom, MagpieData mode GSmagmom, MagpieData minimum SpaceGroupNumber, MagpieData maximum SpaceGroupNumber, MagpieData range SpaceGroupNumber, Mag-

pieData mean SpaceGroupNumber, MagpieData avg-dev SpaceGroupNumber, MagpieData mode SpaceGroupNumber, avg s valence electrons, avg p valence electrons, avg d valence electrons, avg f valence electrons, compound possible, max ionic char, avg ionic char

Supplementary Table 1: Number of model parameters for different DL models

**DL Model**	**#Model Size (MiB)**	**#Model Parameters**
ElemNet	18.6	4,631,361
IRNet	22.0	5,461,473
BNet	14.8	3,670,849
BRNet	18.3	4,548,385

Supplementary Table 2: The table shows the mean ± standard deviation of MAE for different target properties from 5x2-fold cross-validation for all models.

**DL Model**	**Dataset**			
	**OQMD**	**AFLOWLIB**	**MP**	**JARVIS**
ElemNet	0.0736±0.0005	0.0770±0.0002	0.1357±0.0015	0.1128±0.0010
IRNet	0.0640±0.0013	0.0691±0.0008	0.1322±0.0017	0.1040±0.0013
BNet	0.0596±0.0006	0.0655±0.0006	0.1259±0.0015	0.0958±0.0022
BRNet	0.0561±0.0008	0.0625±0.0007	0.1276±0.0018	0.0913±0.0011

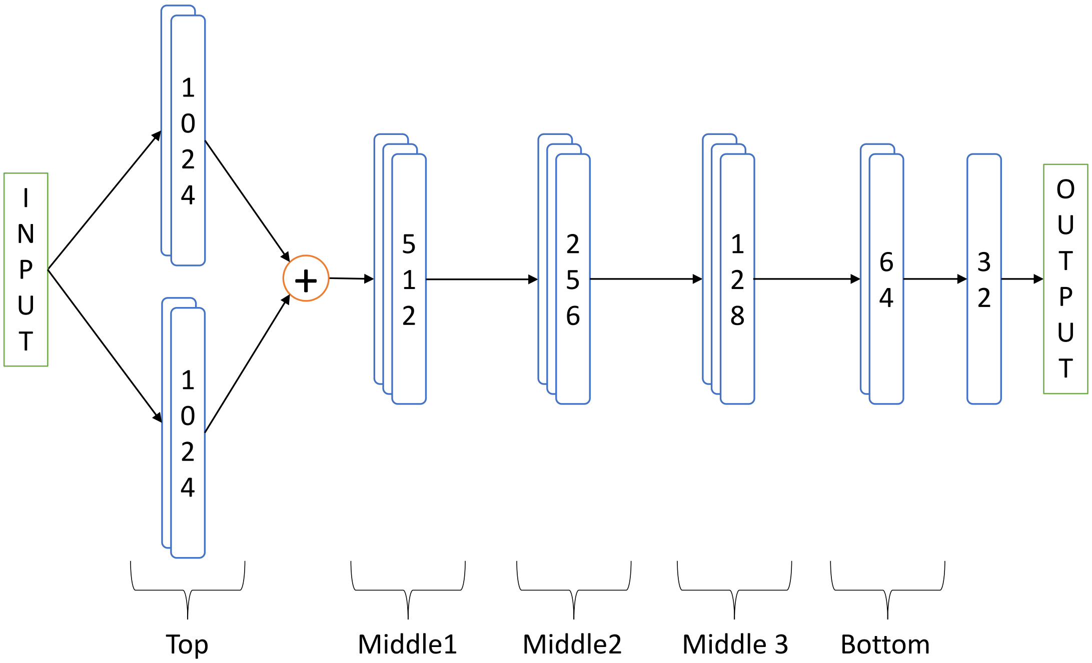

Supplementary Figure 1: Architecture of the DL model (BRNet) used in this work. The stacked structure represents the same layer connected repeatedly.

Mathematical Formulation

A 17-layer neural network is a mathematical function f, which is a composition of multivariate functions: f_1, f_2, ..., f_{16}, and g, defined as:

$$f : \mathbb{R}_n \rightarrow \mathbb{R}_p \tag{1}$$

$$f = g \circ f_{16} \circ f_{15} ... \circ f_2 \circ f_1 \tag{2}$$

where, n is the dimension of the input x, p is the dimension of the output y, g is the output function. Each function f_i is a composed multivariate function defined as follows:

$$f_i(x) = a(w_i x + b_i) \tag{3}$$

where $wx + b$ is a linear combination of the input x with its weight w, a bias b, and a as the activation function (i.e. LeakyReLU=$max(0.01 * x, x)$ in our case). For simplicity, we express the 17-layer neural network as a global function by introducing the weights w and bias b in equation 2 as follows:

$$\begin{aligned} f &= g \circ f_{16} \circ f_{15} ... \circ f_2 \circ f_1 \\ &= g(a(...a(w_2 a(w_1 x + b_1) + b_2) ... + b_{16})) \end{aligned} \tag{4}$$

Now based on the global function shown in equation 4, the global function for different 17-layered neural networks will be defined as follows:

$$\begin{aligned} f^{Gen} &= g(a(...a(w_4 a(w_3 a(w_2 a(w_1 x + b_1) + b_2) + b_3) + b_4) ... + b_{16})) \\ f^{BNet} &= g(a(...a((w_2 a(w_1 x + b_1) + b_2) + (w_4 a(w_3 x + b_3) + b_4)) ... + b_{16})) \\ f^{BRNet} &= g(a(...a((w_2 a(w_1 x + b_1) + b_2) + (w_4 a(w_3 x + b_3) + b_4)) ... + b_{16})) \end{aligned} \tag{5}$$

Here, f^{Gen} is a global function for a straight deep neural network with no or single branching, f^{BNet} is a global function for a branched neural network, and f^{BRNet} is a global function for a branched residual neural network. The composed multivariate function for each of these neural network will be as follows:

$$\begin{aligned} f_i^{Gen} &= a(w_i x + b_i) \\ f_i^{BNet} &= a(w_i x + b_i) \\ f_i^{BRNet} &= x + a(w_i x + b_i) \end{aligned} \tag{6}$$

1. Jha, D. *et al.* ElemNet: Deep learning the chemistry of materials from only elemental composition. *Scientific reports* **8**, 17593 (2018).

2. Ward, L., Agrawal, A., Choudhary, A. & Wolverton, C. A General-Purpose Machine Learning Framework for Predicting Properties of Inorganic Materials. *npj Computational Materials* **2**, 16028 (2016). URL `http://dx.doi.org/10.1038/npjcompumats.2016.28`. `1606.09551`.

3. Ward, L. T. *et al.* Matminer: An open source toolkit for materials data mining. *Computational Materials Science* **152**, 60–69 (2018).
